# “We don’t want to sedate him” - A qualitative interview study on intentions when administering sedative drugs at the end of life in nursing homes and hospitals

**DOI:** 10.1186/s12904-021-00832-0

**Published:** 2021-09-13

**Authors:** Sophie Meesters, Bettina Grüne, Claudia Bausewein, Eva Schildmann

**Affiliations:** Department of Palliative Medicine, University Hospital, LMU Munich, Marchioninistr. 15, 81377 Munich, Germany

**Keywords:** Hospitals; Nursing homes; Palliative Care; Hypnotics and Sedatives; Opioid; Intention; Qualitative research; Deep sedation

## Abstract

**Background:**

Previous data indicate major differences between countries and settings regarding the intention when administering sedative drugs at the end of life and the perception, which drugs are sedating. Therefore, we aimed to explore the concept of ‘sedative drugs’ and the intentions of German healthcare professionals in general palliative care when administering sedative drugs at the end of life.

**Methods:**

Semi-structured qualitative interviews with physicians and nurses (*n* = 49). Recruitment took place via contact persons in five hospital departments (haematology/oncology (*n* = 2), neurology, geriatrics, gynaecology) and five nursing homes. We thematically analysed the transcripts by the Framework approach, using MAXQDA version 2018.2.

**Results:**

Most interviewees referred to benzodiazepines, opioids, and antipsychotics. Some subsumed all into sedative drugs, others differentiated between sedative drugs, anxiolytics, and analgesics. In explaining their intention, interviewees particularly emphasized what they want to avoid when administering sedative drugs. We identified three main themes regarding (excluded) intentions: (1) use of sedative drugs to relieve the patient’s suffering with reduction of consciousness as side effect, (2) use of sedative drugs to relieve the situation for the team and/or the family, (3) distinction between intention and expectation regarding hastening death. Interviewees often equated the term ‘sedation’ with inducing a state of unconsciousness, which should be avoided.

**Conclusion:**

German healthcare professionals in general palliative care seem to negatively connote the term ‘sedation’. Moreover, they see themselves in a more passive role by accepting a side effect rather than performing an intentional act. Critical reflection of indications and intentions in accordance with respective guidelines is needed.

**Supplementary Information:**

The online version contains supplementary material available at 10.1186/s12904-021-00832-0.

## Background

At the end of life, patients may suffer from intolerable symptoms, which are refractory to symptom-oriented treatment. Reducing a patient’s consciousness by administering sedative drugs is an accepted method to relieve suffering [1, 2]. Reduction of consciousness can vary from mild to deep and can be induced intermittently or continuously (until death) [2]. Depending on depth and duration, drug-induced reduction of consciousness can significantly impact on a patient’s social and biological life as well as on the emotional wellbeing of the family and healthcare professionals [3–6]. To reduce insecurities and promote best practice, various guidelines on sedation at the end of life have been developed [7, 8]. However, existing guidelines differ considerably in terminology, definitions and key recommendations [7, 9]. Aspects of consistency between most guidelines include the concept of sedation as intentional reduction of a patient’s consciousness and the recommended drugs [6–8]. However, existing studies indicate major differences between countries and settings regarding the intention of administering sedative drugs [10, 11]. While in some countries healthcare professionals in general palliative care seem to perceive even deep sedation predominantly as side effect of intensified symptom control measures, healthcare professionals in specialist palliative care mostly regard deep sedation as an explicitly intended treatment [11, 12]. Comparing different countries, various studies showed that healthcare professionals in the UK avoid the label ‘sedation’, as they predominantly use low doses of sedatives to make a patient comfortable rather than to sedate him or her. In contrast, healthcare professionals in Belgium and the Netherlands predominantly defined sedation as intentionally reducing consciousness, making sure that patients ‘stayed asleep’ to relieve suffering [10, 13–15]. Moreover, previous data indicate that particularly in general palliative care settings, drugs other than the ones recommended in guidelines are used to reduce a patient’s consciousness at the end of life [16, 17]. The quantitative part of this mixed-methods study revealed that administration of sedative drugs was never labelled as ‘sedation’ in the documents of the participating nursing homes [18]. Given these cultural and setting-specific differences, this study aimed to explore the concept of ‘sedative drugs’ and the intentions of German healthcare professionals in general palliative care when administering sedative drugs at the end of life.

## Methods

### Design, setting, and participants

This qualitative interview study was part of a multicentre mixed-methods study on use of sedatives and sedation at the end of life in general palliative care in Germany. In an explanatory sequential mixed-methods design, a retrospective cohort study was followed by semi-structured qualitative interviews. For this article, only results from the qualitative phase are presented. We conducted interviews with physicians and nurses from hospital departments and nursing homes. Participating hospital departments were haematology/oncology (*n* = 2), geriatrics, gynaecology and neurology of two hospitals (university and teaching hospital). The five participating nursing homes differed regarding affiliation (1 × municipal, 2 × catholic, 2 × protestant), location (4 × city, 1 × suburb), and number of provided beds (102–216). In each participating centre, a contact person was involved for recruitment. Contact persons were provided with information sheets for distribution among their teams. In cases of acceptance, an appointment for a face-to-face interview with the respective team member was made either by email or telephone. GPs were either recruited by the contact persons as they provided care for residents of the participating nursing homes, or they were recruited via professional contacts of the research team. Inclusion criteria were experience in caring for dying patients and sufficient German language skills. Purposive sampling with regard to care setting, profession, gender, age, work experience and palliative care experience was intended to cover a wide range of experiences. However, it was not fully achieved due to difficult recruitment. We followed the COREQ checklist to ensure methodological rigour [19]. For details, see Additional file 1.

The study was approved by the Research Ethics Committee of the Medical Faculty at Ludwig-Maximilians-University Munich (reference number 17–792).

### Data collection

Two researchers (BG, SM) conducted the interviews from May to October 2019 in the interviewees’ workplace or their homes. No one else was present besides the participants and the interviewer. Interview duration ranged between 30 and 81 min with a mean duration of 52 min. Field notes were written after each interview. Parallel to the interviews, the research team constantly discussed whether new and important themes emerged. Interviews were conducted until we assumed data saturation. An interview guide (see Additional file 2) was developed to ensure consistency and adherence to the research questions. It was informed by existing literature as well as the quantitative results of the mixed-methods study that this interview study was part of. The guide covered four main themes: i) Understanding of palliative care and end of life, ii) indications for and intentions of the prescription of sedative drugs, iii) experiences with different types of sedation, and iv) perceived need for change and/or support in handling sedative drugs. We did not define the concept of sedation in advance. To cover experiences with all types of sedation within the interviews, we used a figure illustrating the range from use of sedatives “as needed” via intermittent and/or light to continuous and/or deep sedation. Moreover, we did not specify sedative drugs but asked the interviewees to name drugs they perceived as sedating. The interview guide was pilot tested in five interviews. No repeat interview was necessary. Data on sociodemographic and professional background of the interviewees were collected by a questionnaire. All participants gave their written informed consent. Interviews were audio-recorded and transcribed verbatim, including anonymization.

Both interviewers (BG, SM) have a Master degree in Public health. Initially, both were unexperienced in qualitative research and therefore thoroughly trained and supervised by an experienced qualitative researcher (ES) as well as by external trainings. There were no previous relationships between interviewers and interviewees. Between the project lead (ES) and a few participants, a relationship was already established prior to the project start. In advance to the interview, interviewees were informed about the interviewer’s educational background and occupational status.

### Analysis

We thematically analysed the qualitative interviews by the Framework approach, using MAXQDA version 2018.2 [20]. After familiarization with part of the interview material, we constructed an initial analytical framework, with categories derived both inductively and deductively (close collaboration of SM and BG, with support of ES). The analytical framework was continuously refined during indexing all interviews. At the end of the indexing process, we assumed saturation to be reached as no new themes emerged from the interviews [21]. Therefore, no further interviews were necessary. The analytical framework consisted of eight categories with zero to nine sub-categories, respectively: 1) General information on the treatment at the end of life, 2) Prerequisites and procedure, 3) Understanding of and attitude towards sedation, 4) Experiences with sedation, 5) Interaction and cooperation, 6) Potential for change, 7) Case reports, 8) Other. We summarized and charted the indexed data into a matrix. To ensure consistency, two researchers (SM, BG) independently indexed 16% of the transcripts and summarized a subset of the indexed data. Disagreements were discussed, partly involving a third researcher (ES), until consensus was reached. To address the research question for which the results are reported here, we developed a thematic sheet based on 11 sub-categories of the categories 2, 3, 4, and 6.

We used several strategies to achieve rigor and trustworthiness. Due to anonymization, which was demanded by our data protection officer, transcripts could not be returned to participants. The interviewer, however, continuously confirmed accounts during the interview to guarantee correct understanding. Moreover, we conducted a workshop and final conference for healthcare professionals, including interview participants, where they could provide feedback on the findings. Constant exchange within the project team and repeated discussion with a qualitative expert group at the LMU University Hospital were additional measures to ensure rigour and integrity of analysis.

## Results

Overall, we conducted 49 interviews with the following participants: 12 General Practitioners and 12 nurses in nursing homes; 12 physicians and 13 nurses in hospital departments. The majority of the interviewees was female and 40 years old or older. Median number of years of professional experience was 22, and about half of the participants had training or work experience in specialist palliative care. Characteristics of the interviewees are summarized in Table 1.

**Table 1:** Sociodemographic and professional characteristics of the interviewees

	**Hospital: Nurses** ***n* = 13**	**Hospital:** **Physicians** ***n* = 12**	**Nursing home:** **Nurses** ***n* = 12**	**General practitioners** ***n* = 12**	**Total** ***n* = 49**
**Gender, n (%)**					
Male	2 (15.4)	7 (58.3)	1 (8.3)	8 (66.7)	18 (36.7)
Female	11 (84.6)	5 (41.7)	11 (91.7)	4 (33.3)	31 (63.3)
**Age, n (%)**					
18–29 yeas	2 (15.4)	1 (8.3)	0 (0)	0 (0)	3 (6.1)
30–49 years	8 (61.5)	10 (83.3)	4 (33.3)	3 (25.0)	25 (51.0)
50 +	3 (23.1)	1 (8.3)	8 (66.7)	9 (75.0)	21 (42.9)
**Years of professional experience, median (range)**	17.5 (3.5–35)	7.3 (1–38)	25.5 (9–40)	29.5 (12–45)	22 (1–45)
**Training or work experience in Palliative care (PCU or hospice), n (%)**	8 (61.5)	8 (66.7)	4 (33.3)	6 (50.0)	26 (53.1)
**Hospital speciality, n (%)**					
Neurology	2 (15.4)	3 (25.0)	n/a	n/a	n/a
Haematology/Oncology	6 (46.2)	6 (50.0)	n/a	n/a	n/a
Gynaecology	3 (23.1)	1 (8.3)	n/a	n/a	n/a
Geriatrics	2 (16.7)	2 (16.7)	n/a	n/a	n/a

*n/a* not applicable

### Sedative drugs

Most interviewees referred to benzodiazepines, opioids, and antipsychotics. Few also mentioned sleeping medication, antiemetics, and analgesics in general. Some subsumed all these drugs into the group of sedative drugs. Others differentiated between sedative drugs, anxiolytics, analgesics, and others. The classification of drugs to the groups was individual and not dependent on specific characteristics like setting, profession, or age. Only regarding the use of morphine for reduction of consciousness, three different groups could be distinguished: (1) Predominantly nurses mentioned morphine as sedative drug used for reduction of consciousness, either on its own or combined with other sedative drugs. (2) Especially general practitioners differentiated morphine from sedative drugs but stated to use the sedating side effect of morphine intentionally to reduce consciousness. Accounts from nursing home healthcare professionals showed that states of reduced consciousness in the dying phase are almost entirely induced by subcutaneous morphine injections. (3) Hospital physicians mainly did not consider morphine as sedative drug. They observed the sedating side effect of morphine but did not use it for reduction of consciousness.

### Intention

In explaining their intention, most interviewees particularly emphasized what they want to avoid when administering sedative drugs (excluded intentions). Three main themes were identified regarding the interviewees’ intentions/excluded intentions: (1) use of sedative drugs to relieve the patient’s suffering with reduction of consciousness as side effect, (2) use of sedative drugs to relieve the situation for the team and/or the family, and (3) distinction between intention and expectation regarding hastening death.

*Use of sedatives to relieve the patient’s suffering with reduction of consciousness as side effect: For* most interviewees, regardless of setting and profession, the aim of administering sedative drugs is not reducing the patient’s consciousness. Instead, the intention is to control symptoms and relieve the patient’s suffering or the patient´s situation in general. Doctors and nurses reported that the patient should be calmed or subdued. By “dozing a little” and “snoozing” he is supposed to “relax”, “no longer perceive the situation as bad” and “have the best possible feeling”. Many accounts in this context referred to the dying phase. Especially for nursing home nurses, the use of sedative drugs was reserved to facilitating the dying process and enabling a peaceful dying.

Physicians of both settings reflected that although reduction of consciousness is not the aim, there are situations in which the sedative effect of the drug is a wanted or unwanted side effect to be accepted. A minority mentioned that reduction of consciousness can also be the primary intention but struggled to differentiate when it is the primary intention and when a side effect. Nurses mostly did not differentiate between primary intention and side effect but described administration of sedative drugs solely as means to relieve suffering by calming down the patient. Interviewees used very different terms for the act of reducing consciousness, depending on whether they referred to their intentions or excluded intentions (words italic in quotes).

“Is it now just to *cushion the agitation* or is it really now a full-blown psychosis where I really want to *subdue* the patient significantly. Or do I want to *sedate* him now, so, really just for the purpose of, I don’t know, an MRI. Or for some kind of diagnosis. Therefore, it always depends on the underlying indication” (Helge, hospital physician).

“*Sedation* in itself is rarely a goal. That he must be sedated. It is more the case that the medication we need to *give him some rest*, to *accompany him* somehow on his last journey, is accompanied by sedation.” (Raphaela, hospital physician).

“However, if the patients are highly anxiety-related, then it is, yes, often the case that the drugs we use to *relieve anxiety* also have a sedating element. That is, sedation is a desirable side effect rather than the standard effect […].” (Konrad, general practitioner).

Further statements on the excluded intention to reduce the consciousness were made (Table 2): Healthcare professionals from all settings and professions emphasized that it is never the aim to induce a state of unconsciousness, which was often equated with the term ‘sedation’. For some interviewees, deep sedation constituted a failure in titration, i.e. an overdose. Others described that unconsciousness is generally not intended but can occur during the titration of drugs for symptom control. Reduction or loss of consciousness was mostly perceived as not solely drug-induced but also as a natural effect of the dying process or disease progression. Especially nursing home nurses emphasized that they try to avoid all types of reduction of consciousness. They regarded maintaining the ability to communicate as crucial, and some reported the necessity to accept a certain degree of suffering.

**Table 2:** Avoiding reduction of consciousness

Avoiding states of unconsciousness
Jens, hospital physician “We don’t want to sedate him, we just want to alleviate his suffering and take away his discomfort. We don’t want to euthanize him, but, um, we just want to take away his symptoms […] So that's what you actually want: that he is, um, maybe still able to make contact and somehow maybe still able to express, um, needs. Um, but of course it is not always feasible, and whether that is always due to the medication must of course first be proven.”
Konrad, general practitioner “Intermittent and light, not continuous and deep. Perfectly clear. We are not anaesthetists.”
Nino, hospital nurse “So, it’s not that the patients are completely beamed away, I would say. We don’t do that here.”
Maximiliane, general practitioner B: Some are approachable. […] And then they can still articulate themselves and I don’t have to beat them down
Avoiding all types of reduction of consciousness
Marius, nursing home nurse “As long as they can express themselves, that’s a sign for me that people can still do it. […] And I don’t want to sedate people and free them from everything. They have to be a little confused. They have to be a bit restless, they have to be able to express themselves. And then get the appropriate therapy. But not that they are sleepy and can no longer express themselves.”

*Use of sedative drugs to relieve the situation for the team and/or the family:* Some physicians acknowledged that there might be situations in which sedative drugs are also used to relieve the situation for the team or the family because suffering in dying patients can be difficult to bear. One physician assumed that calming the whole environment is sometimes necessary to ensure a peaceful dying. These physicians reported that it could be challenging to differentiate to what extent the drugs are given in one’s own interest rather than in the patient’s interest. Moreover, some physicians assumed that sedative drugs might sometimes be administered with the intention to reduce care needs.

“I sometimes ask myself: ‘Am I doing this now because I can’t stand it myself somehow?’ So, in a general, figurative sense, when you see a patient suffering so much, that he is always restless, that he is always running around somehow agitated and so on. That is also very, very difficult for the staff, I have to say. […]” (Constanze, hospital, physician).

“She still eats, still drinks and she rings about 90 times a day. Yes? And you can imagine what that means in a nursing ward! As soon as I use sedation medication, she sleeps more and rings less, but otherwise she is always in bed. She is sometimes more, sometimes less responsive. It’s hard, I switch on and off, it’s intermittent.” (Konrad, nursing home, physician).

In contrast, nursing home nurses strictly excluded the intention of tranquilising or restraining a patient by sedative drugs in order to reduce care needs. Medicinal tranquilising or restraining of a patient was perceived to be only acceptable in situations of endangerment of self or others, constituting a deprivation of liberty, which has to be legally covered.

“I only know that a sedation medication is also a deprivation of liberty and that this has to be reported. So, it’s not like that, that you can just give it indiscriminately. […] It is not the case that a person is sedated so that it is easier […].” (Emma, nurse, nursing home).

*Distinction between intention and expectation regarding hastening death:* Although hastening death was not specifically addressed in the interview guide, it emerged as an important intention to be excluded. Predominantly hospital physicians stated that sedative drugs are not and should not be administered with the aim to shorten life, while acknowledging that high doses of sedative drugs might have a life-shortening effect. Although physicians were very clear in their aim not to shorten life, many perceived that the distinction between symptom control and hastening death is a ‘grey area’, in particular when death occurs shortly after drug administration. Physicians reported mainly two arguments to justify the use of sedative drugs despite expecting a possible life-shortening effect: (1) Shortening life for some hours can be justified in the light of relieved suffering and enhanced quality of dying (2) Shortening life can be justified if this risk is clearly communicated and agreed with the patient and/or the family.

In contrast, the majority of nurses clearly distinguished the administration of sedative drugs from hastening death. In their perception, a life-shortening effect can only occur due to errors in handling. In addition, some argued that sedative drugs do not directly hasten death but patients can go more easily and therefore die faster than without medication. Nurses who reflected about a possible life-shortening effect distanced themselves from the responsibility of prescribing.

“Without reaching this terrible situation, which many SAPV services [specialist palliative home care teams] complain about, there is a legal uncertainty regarding the shortening of life and the borderline area between assisted suicide, yes? So do I have/ if you inject morphine subcutaneously in the terminal phase in the case of pulmonary oedema, for example, and severe shortness of breath […] four, five hours later, the patient is dead. Then who can prove to me that I didn’t shorten his life? Nevertheless, of course, it is done. Difficult. It’s not for entrants.” (Konrad, general practitioner).

## Discussion

This qualitative interview study demonstrates that German healthcare professionals in general palliative care predominantly perceive benzodiazepines, antipsychotics, and opioids as sedative drugs. When explaining their intentions, most interviewees additionally emphasized what they want to avoid when administering sedative drugs. Intentions and excluded intentions referred to three main themes: (1) use of sedative drugs to relieve the patient’s situation with reduction of the consciousness as side effect, (2) use of sedative drugs to relieve the situation for the team and/or the family, and (3) distinction between intention and expectation regarding hastening death.

Although it was important for the interviewees to clearly differentiate between intentions and excluded intentions, the line between both seems to be blurred: They want have a dampening effect, but not sedate, they want to calm down, but not tranquilize or restrain, they want to ease the dying process, but not hasten death, and they want to act in the patient’s best interest, but recognize that drugs might also be administered to relieve one’s own situation. Raus and Sterckx regarded the assumption of a single and clear-cut intention of sedation as oversimplification, reflecting the described balancing act between different intentions and excluded intentions [22]. Moreover, it is difficult to externally verify the intention of an act, which may contribute to our interviewees very cautious handling of sedative drugs which we will report elsewhere [22, 23].

Our finding that healthcare professionals mostly perceive reduction of consciousness as a side effect of drugs used to relieve the patient’s suffering at the end of life is in accordance with studies from the UK, Switzerland and the US and contrary to studies from the Netherlands and Belgium [10–13, 15]. According to our interviewees, they almost never use sedation intentionally. This is probably related to seemingly negative connotations of the term ‘sedation’. Our interviewees predominantly used the term ‘sedation’ for inducing unconsciousness, and partly associated it with deprivation of liberty or hastening death. As these aspects were all reported to be excluded intentions, the interviewees´ avoidance of the term ‘sedation’ seems the logical consequence. Interviewees even labelled intentional reduction of consciousness as a means to relieve suffering as “wanted side effect” rather than ‘sedation’. Accordingly, they seem to assign themselves a more passive role by accepting a side effect rather than acting intentionally, possibly to (subconsciously) reduce responsibility and legal accountability for the treatment and its consequences.

Avoidance of the term ‘sedation’ for use of sedative drugs resulting in reduction of consciousness has important consequences. Healthcare professionals who do not perceive and label their treatment as ‘sedation’ will not refer to respective guidelines, even if they are aware of them. This may be one of the reasons for deviations from guidelines regarding sedation, which have been reported particularly for the general palliative care setting, for example regarding indications, processes of decision-making and choice of drugs – including the use of opioids for sedation [17, 18, 24].

All interviewed healthcare professionals emphasized their intention to relieve the patient’s suffering while maintaining the ability to communicate as far as possible. Especially nurses from nursing homes emphasized that they try to avoid any reduction of consciousness, even accepting suffering to a certain extent. So far, differences regarding the value of consciousness have been found between countries and between healthcare professionals [10, 25]. Our results indicate that healthcare setting, in this case nursing homes, can additionally influence the importance of consciousness in balancing it against suffering. Most probably, this result is associated with the public scrutiny, under which the use of sedative drugs specifically in German nursing homes is placed [26, 27].

Various studies have demonstrated anxieties among healthcare professionals to hasten death by the use of sedative drugs [10, 11, 28, 29]. Interestingly and in contrast to previous findings, respective considerations were mainly voiced by physicians in our study, while nurses only rarely raised this issue or clearly distinguished using sedative drugs from hastening death [10, 28]. This inter-professional difference may contribute to disagreements regarding timing and dose of sedative drugs described by our interviewees, which we will report elsewhere: Nurses’ fewer concerns regarding a potential life-shortening effect may trigger their perception of physicians’ use of sedative drugs as too cautious and too late. Contrary to other studies among general palliative care professionals, our interviewees clearly excluded the intention to hasten death [12, 13, 30]. Instead, and in line with findings from specialist palliative care, the physicians in our study emphasized the distinction between intention and expectation regarding hastening death with acceptance of a possible life-shortening effect only in the dying phase [31].

### Limitations & strengths

The research team was aware of the risk of social desirability bias regarding the sensitive issue of sedation, and tried to minimise it by the information provided prior to and during the interview, the interview guide and training of the interviewers. Despite our efforts of purposive sampling, including specific motivation of contact persons to recruit inexperienced healthcare professionals, the latter were underrepresented. Their perspectives may therefore not be fully taken into account. Moreover, we did not receive any information on reasons for non-participation. However, the main strength of the study is the inclusion of nurses and physicians of different hospital departments as well as nursing homes, covering a large range of experiences and perspectives. The diverse sample allowed comparisons between settings as well as between professions.

## Conclusion

Education in general palliative care should focus more on the differentiation and potential transition from sedation as a side effect to intentional sedation – which includes also light and/or intermittent sedation. Moreover, it is important to overcome negative associations with the term ‘sedation’ and reduce insecurities regarding intentional sedation. First, appropriate labelling of the treatment by healthcare professionals is the prerequisite for using respective guidelines and therefore promotion of best practice. Second, reduction of consciousness should be explicitly decided for and adequately labelled to enable clear and transparent communication within the team as well as with patients and families.

## Supplementary Information

**Additional file 1.** COREQ Reporting Checklist

**Additional file 2.** SedEol - Interview guide for hospital departments – nurses*

## Data Availability

The datasets generated and/or analysed during the current study are not publicly available due to ensuring data protection and anonymity for the interviewees but are available from the corresponding author on reasonable request.
