# Supplementary material for: “We don’t want to sedate him” - A qualitative interview study on intentions when administering sedative drugs at the end of life in nursing homes and hospitals
Source: BMC Palliat Care. 2021 Sep 13;20:141. doi: 10.1186/s12904-021-00832-0 (PMC8439055; doi:10.1186/s12904-021-00832-0)
Supplement: Supplementary file 1 — Additional file 1. COREQ Reporting Checklist [file 12904_2021_832_MOESM1_ESM.docx]

**COREQ Reporting Checklist**

| Domain 1: Research team and reflexivity | |
| --- | --- |
| Personal Characteristics | |
| 1. Interviewer/facilitator | Bettina Grüne (BG), Sophie Meesters (SM), detailed in methods section “Data collection” (p5) |
| 1. Credentials | BG: Master of science, Dr. rer. biol. hum.  SM: Master of Public Health  Eva Schildmann (ES): MD/Dr. med., Master of Science  Claudia Bausewein (CB): PhD/Professor, MD/Dr. med., Master of Science |
| 1. Occupation | BG, SM: research associates  ES: physician and leader of research group, leader of this project  CB: physician and head of department |
| 1. Gender | BG, SM, ES, CB: Female |
| 1. Experience and training | Reported in the methods section “Data collection” (p5):  Additional information: ES conceptualized and realized several qualitative interview studies. |
| Relationship with participants | |
| 1. Relationship established | Reported in the methods section “Data collection” (p5)  Additional information: Relationships were of a neutral to friendly professional character. |
| 1. Participant knowledge of the interviewer | Reported in the methods section “Data collection” (p5-6)  Additional information: information included that they had no clinical experience. |
| 1. Interviewer Characteristics | Reported in the methods section “Data collection” (p5)  Additional information: The interviewers have a research interest in health care research, especially in use of sedating drugs at the end of life in general palliative care. |
| Domain 2: Study design | |
| Theoretical Framework | |
| 1. Methodological orientation and theory | Stated in the methods section “Analysis” (p6): Data were analysed using the Framework approach. |
| Participant selection | |
| 1. Sampling | Stated in the methods section “Design, setting, and participants” (p4). |
| 1. Method of approach | Stated in the methods section “Design, setting, and participants”.  (p4). |
| 1. Sample size | Reported in the first paragraph of the results section (p7): 24 physicians, 25 nurses |
| 1. Non-participation | Reported in the Discussion section “Limitations & strengths”  (p15). |
| Setting | |
| 1. Setting of data collection | Reported in the methods section “Data collection”  (p5). |
| 1. Presence of non-participants | Reported in the methods section “Data collection”  (p5). |
| 1. Description of Sample | Reported in the first paragraph of the results section (p7). |
| Data Collection | |
| 1. Interview Guideline | The development of the interview guide is briefly described in the methods section “Data collection” (p5).  Additional information: The development followed the prodecure according to Helfferich [1]. The interview guide was pilot tested in five interviews. Due to only minor changes in the guide, those interviews were also included in analyses. |
| 1. Repeat interviews | Reported in the methods section “Data collection”  (p5). |
| 1. Audio/Visual recording | Reported in the methods section “Data collection” (p5). |
| 1. Field notes | Reported in the methods section “Data collection” (p5)  Additional information: A field-note form was filled in by the researcher after each interview, covering the following topics: relationship between researcher and interviewee, interview setting, account on interruptions, interview atmosphere, perceived moods or emotions of interviewee, difficulties in carrying out the interview, comments on content, feelings of the researcher. In case of a distinctive behaviour or strong emphasizing on certain topics, respective memos were added to the transcript. |
| 1. Duration | Reported in the methods section “Data collection” (p5) |
| 1. Data saturation | Reported in the methods section “Data collection” (p5): Parallel to the interviews, the research team discussed constantly whether new and important themes emerged. Interviews were conducted until we perceived data saturation.  Reported in the methods section “Analysis” (p6): At the end of the indexing process, we found saturation to be reached as no new themes emerged from the interviews. Therefore, no further interviews were necessary. |
| 1. Transcripts returned | Reported in the methods section “Analysis” (p6): Due to anonymization, transcripts could not be returned to participants. The interviewer, however, continuously confirmed accounts during the interview in order to guarantee correct understanding. |
| Domain 3: Analysis and findings | |
| Data analysis | |
| 1. Number of data coders | Reported in the last paragraph of the methods section “Analysis” (p6). |
| 1. Description of the coding tree | A coding tree and a coding guide with detailed descriptions was developed by the coders to ensure quality. It is available from the authors on request (in German language). |
| 1. Derivation of themes | Reported in the methods section “Analysis” (p6): inductive as well as deductive approach |
| 1. Software | Reported in the methods section “Analysis” (p6): MAXQDA version 2018.2 |
| 1. Participant checking | Reported in the methods section “Analysis” (p6): We conducted a workshop and final conference for healthcare professionals, including interview participants, where they could provide feedback on the findings. |
| Reporting | |
| 1. Quotations presented | Quotations from different participants are presented to illustrate the findings, and each quotation is identified by a name (no relation to the real name of the participants). |
| 1. Data and findings consistent | Yes |
| 1. Clarity of major themes | The concept of sedative drugs and intentions when administering sedative drugs emerged as an important aspect in the context of the broader research interest “administration of sedating drugs and sedation”. We presented the major themes regarding the concept and intentions of administering sedative drugs. |
| 1. Clarity of minor themes | As far as of interest for our research aim, we also presented minor themes. |

[1]: Helfferich, C., [The Quality of Qualitative Data – Manual for the Implementation of Qualitative Interviews] Die Qualität qualitativer Daten – Manual für die Durchführung qualitativer Interviews. 4. edition 2011, Wiesbaden: VS Verlag für Sozialwissenschaften |Springer
