# Supplementary material for: “We don’t want to sedate him” - A qualitative interview study on intentions when administering sedative drugs at the end of life in nursing homes and hospitals
Source: BMC Palliat Care. 2021 Sep 13;20:141. doi: 10.1186/s12904-021-00832-0 (PMC8439055; doi:10.1186/s12904-021-00832-0)
Supplement: Supplementary file 2 — Additional file 2. SedEol - Interview guide for hospital departments – nurses* [file 12904_2021_832_MOESM2_ESM.docx]

| **Central question** | **Checks for further questions** | |
| --- | --- | --- |
| 1. Last year we went to different hospital wards and looked at the medical records of deceased patients. In the records, the term "palliative", e.g. "palliative treatment", "change of treatment aim with palliative symptom control", was often documented.   When do you use the term palliative treatment? | Understanding of “palliative”  Understanding of the end of life  Influence on changes in medication | |
| 1. In your experience, when are sedating drugs administered at the end of life?   Example: drugs which make you sleepy or drowsy (i.e. which reduce consciousness), for example tranquilliser such as lorazepam | Indications/criteria for sedating drugs at the end of life  Aims/Intention when administering drugs  Which drugs are used?  Further probing, only if mentioned by the interviewee:   - Intolerable suffering - Physical vs. psychological symptoms - Refractory symptoms | |
|  | **Central question** | **Checks for further questions** |
| **Figure I**  **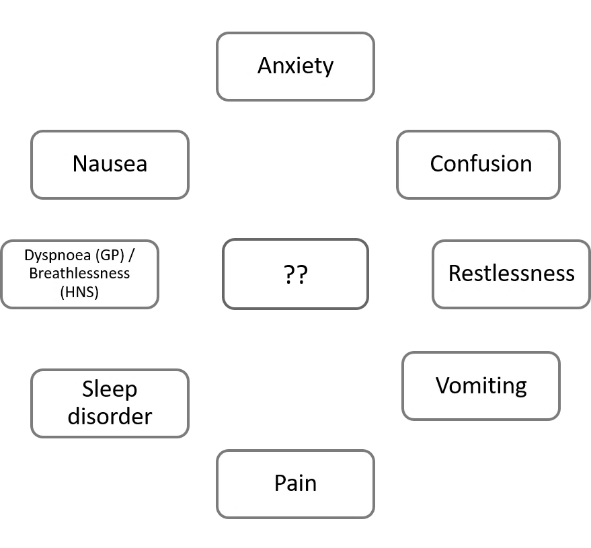** | 1. These are some of the symptoms for which sedating drugs were given according to the medical records (see figure I). What do you think about them? | Appropriate use  Kind of drugs used for these symptoms  Missing symptoms/indications for sedating drugs  Inappropriate use |
| 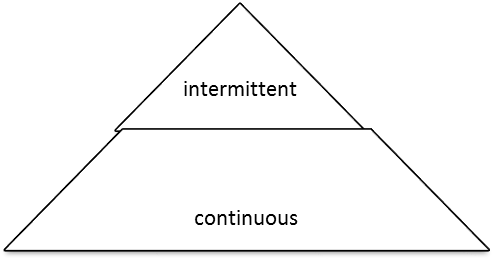 Deep sleep/ Unconsciousness  Mild Sleepiness/ Slightly reduced consciousness  **Figure II**  In the medical records, we could see that the administration of these drugs can lead to mild sleepiness/a slightly reduced consciousness in some patients and to deep sleep/unconsciousness in others. The medication was sometimes given intermittently, e.g. only on one day, and sometimes it was given continuously, e.g. over several days. | | |
| **Central question** | **Checks for further questions** | |
| 1. What are your experiences with these different types of treatment at the end of life? | General experiences  Good aspects  Uncertainties/challenges  Professional support  Cooperation with specialist palliative care services  Description of a specific case | |
| **Central question** | | |
| 1. Finally, if you think about your previous experiences with sedation at the end of life   A) Is there anything that should be changed?  B) What could help you in your everyday work? | | |
| 1. Is there anything else you would like to mention? | | |

*Interview guides were developed for nursing home nurses, nursing home physicians as well as for hospital nurses and hospital physicians, with slight adaptions, respectively.
